# The mechanism of potato resistance to *Globodera rostochiensis*: comparison of root transcriptomes of resistant and susceptible *Solanum phureja* genotypes

**DOI:** 10.1186/s12870-020-02334-2

**Published:** 2020-10-14

**Authors:** Alex V. Kochetov, Anastasiya A. Egorova, Anastasiya Y. Glagoleva, Kseniya V. Strygina, Elena K. Khlestkina, Sophia V. Gerasimova, Natalja V. Shatskaya, Gennady V. Vasilyev, Dmitry A. Afonnikov, Nikolay A. Shmakov, Olga Y. Antonova, Natalia V. Alpatyeva, Alexander Khiutti, Olga S. Afanasenko, Tatjana A. Gavrilenko

**Affiliations:** 1 Institute of Cytology and Genetics, SB RAS, Novosibirsk, 630090 Russia; 2 Novosibirsk State University, Novosibirsk, 630090 Russia; 3 Vavilov Institute of Plant Genetic Resources (VIR), Saint Petersburg, 190000 Russia; 4 All Russian Research Institute for Plant Protection, Saint Petersburg, 196608 Russia

**Keywords:** *Solanum phureja*, Nematode, Resistance, *Globodera rostochiensis*, Transcriptome, Potato

## Abstract

**Background:**

*Globodera rostochiensis* belongs to major potato pathogens with a sophisticated mechanism of interaction with roots of the host plants. Resistance of commercial varieties is commonly based on specific R genes introgressed from natural populations of related wild species and from native potato varieties grown in the Andean highlands. Investigation of molecular resistance mechanisms and screening the natural populations for novel R genes are important for both fundamental knowledge on plant pathogen interactions and breeding for durable resistance. Here we exploited the *Solanum phureja* accessions collected in South America with contrasting resistance to *G. rostochiensis*.

**Results:**

The infestation of *S. phureja* with *G. rostochiensis* juveniles resulted in wounding stress followed by activation of cell division and tissue regeneration processes. Unlike the susceptible *S. phureja* genotype, the resistant accession reacted by rapid induction of variety of stress response related genes. This chain of molecular events accompanies the hypersensitive response at the juveniles’ invasion sites and provides high-level resistance. Transcriptomic analysis also revealed considerable differences between the analyzed *S. phureja* genotypes and the reference genome.

**Conclusion:**

The molecular processes in plant roots associated with changes in gene expression patterns in response to *G. rostochiensis* infestation and establishment of either resistant or susceptible phenotypes are discussed. De novo transcriptome assembling is considered as an important tool for discovery of novel resistance traits in *S. phureja* accessions.

## Background

Potato cyst nematodes originated in Andean regions of South America [1]. The Golden Potato Cyst Nematode (GPCN, *Globodera rostochiensis*) is a quarantine pest found worldwide and responsible for considerable losses in potato production [2, 3]. At present, *G. rostochiensis* (pathotype Ro1) was registered in 61 territorial entities of the Russian Federation, including 861 administrative districts covering an area of 1,794,442 ha [4, 5]*.* Depending on the potato cultivar, yield losses can range from 19 to 90% [6], and GPCN eggs can remain dormant and viable inside the cyst for 30 years [7]. The predicted impact of *G. rostochiensis* may also grow due to the climate change [8].

The protection of plants against potato cyst nematodes is complicated and commonly involves the usage of nematicides [8, 9] and trap crops (e.g., *Solanum sisymbriifolium*) [3, 10]. However, many chemical nematicides are either low efficient [11, 12] or toxic and prohibited in Europe, and the control of GPCN is mainly based on deployment of major resistance genes (R-genes) (e.g., [13]). R genes conferring strong resistance to the pathotype Ro1 of GPCN were introgressed into commercial varieties from South America originated species: the *H1* gene from the cultivated species *Solanum tuberosum subsp. andigenum* [14], and the *Gro1–4* gene from the Bolivian wild species *S. spegazzinii* [15, 16]. It is widely discussed that introgression of new R genes is of importance since there is a threat of nematode evolving [13, 17].

The GPCN juveniles penetrate plant roots in which they induce the formation of feeding syncytia. The effector proteins from oesophageal glands trigger the chain of metabolic and morphological changes resulting in fusion of several root cells into a feeding structure as well as suppression of plant immune response [18]. In a few investigations of plant varieties resistant to sedentary nematodes the revealed resistance mechanisms were mainly based on incompatible interaction and hypersensitive response: a zone of dead cells formed either at early or at later stages after infestation isolated juveniles from nutrient supply and prevented completion of their life cycle [19]. The natural variety of resistance mechanisms against potato sedentary nematodes has not been evaluated yet. Recent studies highlighted the biological complexity of plant-nematode interactions, for example the role of unusual R genes (such as soybean Rhg1 alpha-SNAP proteins efficient against a wide variety of cyst nematodes) [20, 21], new nematode effectors and related mechanisms of immunity suppression (e.g., *RHA1B*) [22].

Both compatible and incompatible plant-nematode interactions provide unique models for detailed investigation of fundamental genetic mechanisms of plant cell reprogramming, its prevention by defense systems and host-pathogen coevolution. The process of interaction is rather complicated: an important feature of nematode inoculation is significant tissue damage resulting in a non-specific wounding stress. This non-specific wounding response may overlap with the specific response to GPCN or be an integral part of it [23].

Here we present the results of detailed comparative analysis of root transcriptomes of two diploid potato accessions of *S. phureja* with contrasting resistance to GPCN. The dynamics of transcriptome changes after nematode juveniles inoculation revealed a combination of biological processes related to cell division, tissue regeneration, non-specific defense against pathogens, as well as some processes with an unknown role (e.g., changes in plastid associated metabolic chains). The molecular mechanisms of nematode propagation and plant resistance are discussed. Another observation concerns the general approaches of studying the accessions of Andean native potato varieties. It was found that the reference genome annotation is not sufficient for analysis of transcriptomes of these cultivated diploid potato accessions from VIR collection and de novo transcriptome assembling may be used to fill this gap (at least, partially). One of the aims of this study was to compile a set of new candidate R genes against GPCN for further investigation and introgression through either conventional breeding or other biotechnological approaches (e.g., [24–28]).

## Results

### Comparison of transcriptomes of GPCN-resistant and susceptible *S. phureja* genotypes

The samples of roots of *S. phureja* accessions i-0144786 and i-0144787 were taken before inoculation (0), 24 and 72 h post inoculation (hpi) with either GPCN juveniles or water (3 plants per time point / type of treatment). Thirty libraries of paired reads consisting of 1,265,033,228 paired reads were obtained as raw sequencing data. After filtering, 967,259,304 paired reads remained as clean sequencing data. Average library size was about 42 million read pairs before and 32 million read pairs after filtering (Additional file 1).

On average, 65% of read pairs were mapped concordantly to the reference genome [29] with the aid of STAR tool. Read counts were computed for all the annotated 39,021 genes. Filtering of low-expression genes prior to differential expression evaluation resulted in a sample of 21,113 genes for further analysis.

**Fig. 1:**
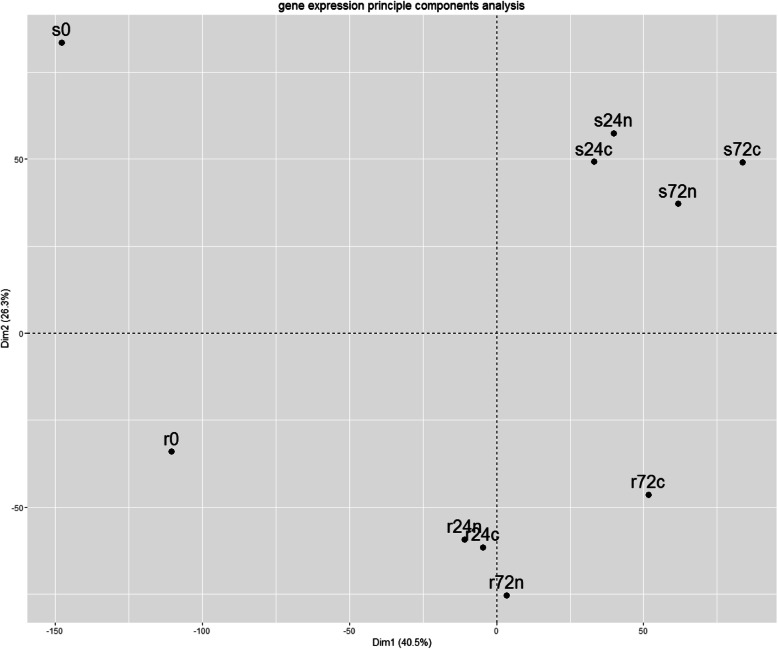
PCA plot of samples analyzed for two largest components. The first component, X axis (40.5% of total variance), the second component, Y axis (26.3% of total variance). Designations: r, *S. phureja* genotype resistant to GPCN; s, *S. phureja* genotype susceptible to GPCN; 24, 72 – hours after plant roots inoculation (hpi) with either water (c, control) or nematode (n); 0, samples of resistant and susceptible plants roots without any inoculation. For example, r24n, transcriptome of *S. phureja* resistant genotype taken at 24 hpi point

The difference between the transcriptomes of two compared *S. phureja* genotypes was evaluated with principal component analysis (PCA, Fig. 1). The first principal component (axis X) corresponds to changes in gene expression patterns at different time points (0, 24, 72 hpi) for both resistant and susceptible genotypes. The second component (Y axis) characterizes the differences in gene expression patterns between the two analyzed genotypes.

**Fig. 2:**
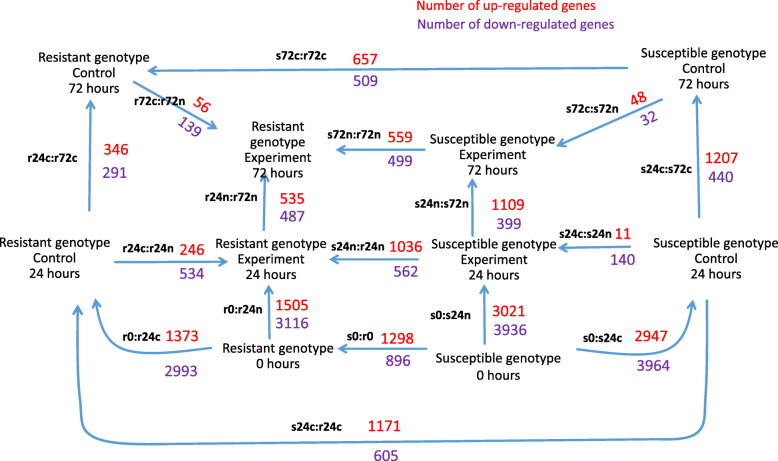
The numbers of DEGs in *S. phureja* roots sampled under various experimental conditions. The arrow shows the compared transcriptomes, the numbers of up regulated genes are shown in red, the numbers of down-regulated genes are shown in purple. Designations: control, water inoculation; experiment, nematode inoculation; r, resistant genotype; s, susceptible genotype; 24, or 72 – hours after plant roots inoculation with either water (c, control) or nematode (n); 0, samples of roots of resistant and susceptible genotypes collected without inoculation. For example, s24c:r24c means comparison of transcriptomes from susceptible (s) and resistant (r) *S. phureja* genotypes collected 24 h (24) after inoculation with water (c)

One may see that (i) the *S. phureja* GPCN-resistant and susceptible genotypes have different transcriptomes that may refer to different origins of these accessions; (ii) the largest changes in gene expression patterns occur at 24 hpi, the subsequent changes at 72 hpi are relatively smaller; (iii) changes in transcriptomes were more pronounced in susceptible plants than in resistant genotype. Numbers of DEGs were calculated for the following pairwise comparisons: (I) to reveal the genes up- or down-regulated in susceptible genotype with respect to the resistant sample under the same experimental conditions (s0:r0, s24n:r24n, s24c:r24c, s72n:r72n, s72c:r72c); (ii) to reveal the transcriptome differences in the plants of one genotype at different hpi points (r0:r24c, r24c:r72c, r0:r24n, r24n:r72n, s0:s24c, s24c:s72c, s0:s24n, s24n:s72n); (iii) to reveal the transcriptome differences in plants of the same genotype but inoculated with either water (c, control) or nematode (n) (r24c:r24n, r72c:r72n, s24c:s24n, s72c:s72n). Overall, it resulted in 17 pairwise comparisons (Fig. 2).

The diagram (Fig. 2) reflects the numbers of identified DEGs. The arrows show the direction of comparison. For example, comparison s0:r0 between susceptible genotype at the 0 h (s0) and resistant accession at the same time (r0) yields 1298 genes which expression levels are higher and 896 genes which expression levels are lower than in resistant genotype. Full lists of differentially expressed genes are available as Additional file 2.

These data coincided with PCA plot (Fig. 1). First, the number of DEGs is greater between the root transcriptomes at 0 and 24 hpi, than between 24 and 72 hpi for both genotypes. The numbers of DEGs between the roots of resistant genotype after inoculation with water at 0 and 24 hpi were 1373 (up) and 2993(down), whereas for 24 hpi and 72 hpi the numbers of DEGs were 346 (up) and 291 (down). A similar situation was observed for resistant *S. phureja* accession after nematode inoculation: r0:r24n (1505 (up), 3116 (down)) versus r24n:r72n (535 (up), 487(down)). Second, the number of DEGs was generally larger in the roots of susceptible cultivar. Finally, the roots of susceptible plants were characterized by a relatively small difference in DEGs after inoculation with either water or nematode juveniles. For example, s24c:s24n comparison revealed 11 up- and 140 down-regulated genes, whereas comparison of root transcriptomes of *S. phureja* resistant plants after inoculation with either GPCN or water (r24c:r24n) revealed 246 up- and 534 down-regulated genes.

### Gene ontology

The results of gene sets enrichment analyses for different plant sample pairs were obtained with the aid of AgriGO (Additional file 3 contains the full lists of GO terms for all the comparisons). Summary of observed trends is presented in Table 1.

**Table 1:** Summary of AgriGO analysis of root transcriptomes of resistant and susceptible *S. phureja* genotypes

Pairwise comparison of genotypes	Upregulated genes & associated processes	Downregulated genes & associated processes
Resistant vs Susceptible (control point, 0 hpi)	Oxidation reduction and associated terms.	Signaling, phosphorylation and associated terms. Oxidoreductase activity (small group of DEGs)
Resistant at 24 hpi (water) vs Resistant before inoculation	Regeneration after wounding (cell cycle, mitosis, etc.)	Signaling, protein modification and associated terms probably reflecting metabolism reprogramming toward the stress response.
Resistant at 24 hpi (GPCN) vs Resistant before inoculation	Variety of stress-response related terms (responses to stimuli, oxidative stress, peroxidase activity, glycosylases, endopeptidase inhibitors, etc.) Photosynthesis-related genes (probably related to organelle division in the frame of cell division and elongation processes).	Signaling, protein modification and associated terms probably reflecting metabolism reprogramming toward the stress response.
Resistant at 72 hpi (water) vs Resistant at 24 hpi (water)	Small changes in spectrum of oxidoreductases. Nothing special at a large scale.	Reproduction, oxidation reduction, small changes in spectrum of oxidoreductases. Nothing special at a large scale.
Resistant at 72 hpi (GPCN) vs Resistant at 24 hpi (GPCN)	Variety of stress-response related terms (oxidation reduction, peroxidase activity, glycosylases, endopeptidases, proteases, ribonucleases, etc.) Cell wall biogenesis (cell division, elongation of cells during regeneration, or syncytium development)	Photosynthesis, significant changes in metabolic processes, changes in oxidoreductases spectra.
Susceptible at 24 hpi (water) vs Susceptible before inoculation (0 hpi)	Response to various stimuli, cell cycle. Photosynthesis (probably related to plastid division in the frame of tissue regeneration after wounding or some changes in plastid metabolism).	Signaling, protein modification and associated terms. Downregulation of a wide variety of regulatory and metabolic processes possibly reflecting metabolism reprogramming toward the stress response.
Susceptible at 24 hpi (GPCN) vs Susceptible before inoculation (0 hpi)	Cell cycle and related terms. Response to stimuli.	Signal transduction, downregulation of a wide variety of regulatory and metabolic processes.
Susceptible at 72 hpi (water) vs Susceptible at 24 hpi (water)	Cell cycle and related terms (mitosis, microtubule-based movement, etc.). Changes in spectra of oxidoreductases and hydrolyses activities, probably associated with metabolic reprogramming.	Small changes in signaling and oxidoreductases – associated activities.
Susceptible at 72 hpi (GPCN) vs Susceptible at 24 hpi (GPCN)	Cell cycle-related terms. Terms related to activation of metabolic processes (positive regulations of variety of activities). Various peptidase activities.	Small changes in oxidoreductases – associated activities, signal transduction, response to stimuli.
Resistant at 24 hpi (water) vs Susceptible at 24 hpi (water)	Cell cycle related terms. Response to stimuli. Stress response related terms (peroxidase activity, oxidoreductase activity, hydrolase activities). Plastid related terms. Higher expression level of some general metabolism-related genes (e.g., catalytic activity)	Nothing specific at large scale (e.g., photosynthesis related terms, response to stimuli, etc.)
Resistant at 72 hpi (water) vs Susceptible at 72 hpi (water)	Oxidation reduction related terms. Nothing special at a large scale.	Nothing specific at large scale (e.g., photosynthesis related terms)
Resistant at 24 hpi (GPCN) vs Susceptible at 24 hpi (GPCN)	Oxidation reduction, oxidoreductases – associated terms. Higher expression levels of photosynthesis related genes (probably reflecting changes in plastids). Peptidase inhibitor activity.	Nothing specific at large scale. Some changes in oxidoreductases – associated activities
Resistant at 72 hpi (GPCN) vs Susceptible at 72 hpi (GPCN)	Nothing specific at large scale. Some changes in oxidoreductases – associated activities. Peptidase inhibitor activity.	Nothing specific at large scale. Some changes in photosynthesis and oxidoreductases – associated activities, some peptidase inhibitor activities.

#### De novo transcriptomes assembly

*S. phureja* genotypes under investigation were collected in South America and their genomes may differ from the reference genome [29]. Thus, annotation of transcriptome based only on the alignment with the reference genome is not complete and some important genes can be missed from consideration. To fill this gap at least partially, we additionally studied the *S. phureja* transcriptomes constructed de novo without usage of the reference genome. For this purpose, two master-transcriptomes were assembled. Raw transcriptome assemblies consisted of 538,200 (resistant accession i-0144787) and 643,926 contigs (susceptible accession i-0144786). After reducing redundancy of assemblies with Evidential Gene software, 168,254 and 172,210 contigs remained in non-redundant assemblies of resistant and susceptible *S. phureja* transcriptomes, respectively. BUSCO analysis revealed 89.9% of complete and 6.9% of fragmented BUSCOs for resistant and 74.4% of complete and 13.3% of fragmented BUSCOs for susceptible genotype.

On average, 78% of clean read pairs were aligned to the non-redundant sets of contigs of resistant variety and 72% of susceptible variety. Furthermore, non-redundant sets of contigs were aligned against reference genome, CDS and protein sequences. The results of TPM filtering and alignments of contigs to the reference genome and CDS sequences are summarized in Table 2.

**Table 2:** Numbers of contigs identified in de novo transcriptome assembly of susceptible and resistant *S. phureja* genotypes at various TPM threshold values

TPM threshold	Susceptible genotype			Resistant genotype		
	Total contigs	Novel contigs	novel contigs with predicted Pfam domains	Total contigs	Novel contigs	novel contigs with predicted Pfam domains
1	66,664	7915	4817	54,866	11,269	6017
2	53,094	4233	2520	41,481	6139	3249
3	46,289	3038	1757	35,620	4571	2338
4	41,682	2424	1370	31,810	3795	1889
5	38,152	2025	1131	28,950	3301	1593

One may see that there is a pool of novel transcripts with no significant homology to the reference genome (e-value defined by standalone blastn software e < 10^− 30^). Indeed, functions of these novel transcripts cannot be predicted through alignment with the reference genome and their functional analysis is in need of other approaches. Thus, we made a prediction of domains in proteins potentially encoded by de novo assembled contigs with the aid of HMM package (the results of contigs alignment to CDS and genome as well as pfam domains predictions are available in Additional file 4). It was found that a considerable part of the novel transcriptome encodes polypeptides with homology to Pfam-annotated functional domains, i.e. these mRNAs potentially encode functional proteins (Table 2).

Indeed, transcriptome analysis needs additional support to verify the expression of potential functional transcript at the level of translatome and proteome (e.g., [30–32]). However, the data presented provides a background for further experimental investigation of particular genes of interest, e.g., those encoding NBS-LRR receptors. It is well known that R-genes providing resistance to GPCN commonly belong to NBS-LRR receptor family. It has been reported earlier that the resistance of i-0144787 *S. phureja* accession did not result from the presence of already known R genes [4]. Since some de novo assembled transcripts have no counterparts in the reference genome annotation, the search for novel R-genes should be expanded on these contigs. To reveal the potential NBS-LRR genes responsible for GPCN resistance, we characterize de novo assembled transcripts with the aid of NLR-Parser software [33]. It was found that a number of cDNAs encode proteins with NBS-LRR domains, and some of these contigs were either not annotated or even have no homology with the reference genome. For susceptible *S. phureja* variety, 30 contigs were predicted as NBS-LRR gene candidates and all of them can be aligned onto the reference genome, but only 26 were aligned to genomic loci with annotated protein coding genes (i.e., four cDNA are likely to be encoded by genes missed from annotation). For GPCN-resistant *S. phureja* genotype, 33 cDNAs encoding NBS-LRR proteins were predicted. Thirty-two contigs can be aligned onto the reference genomic sequence, and 28 of them were mapped to known protein-coding loci. Thus, the transcriptomes of both *S. phureja* accessions contain additional transcripts of novel NBS-LRR encoding genes (Additional file 4). Interestingly, one contig predicted as NBS-LRR encoding cDNA by NLR-Parser, has no homology to the reference genome or CDS sequences at nucleotide sequence level. It has a size of 3112 nucleotides, and its predicted translated product is 865 amino acids long. The contig has an NB-ARC domain predicted with significance value E = 1.1∙10^− 39^ and LRR-8 domain predicted with E = 4.5∙10^− 6^. Alignment of the contig to NCBI non-redundant gene sequences set showed homology to five entries listed in Table 3.

**Table 3:** Novel NBS-LRR gene in *S. phureja* GPCN-resistant genotype has no homology in the reference genome but with some other Solanaceae species

Accession	Organism	Identity,%	alignment size, n
XM_015213980.1	*Solanum penellii*	92	2614
XM_004234191.4	*S. lycopersicum*	91	2672
CU468268.3	*S. lycopersicum*	92	1713
XM_009787721.1	*Nicotiana sylvestris*	76	2139
XM_009787714.1	*N. sylvestris*	76	2139

In addition, this cDNA contig has homology to the nucleotide sequences in chromosomes 3 of *S. lycopersicum* and *S. penellii*. At the same time, amino acid sequence of the contig’s predicted translated product showed homology to a number of protein sequences presented in NCBI Protein database. Interestingly, among 100 sequences with best hits there were no sequences from *S. tuberosum* or *S. phureja*. Ten best hits include sequences of *S. pennellii*, *S. lycopersicum*, *N. tabacum*, *N. tomentosiformis*, and *N. sylvestris*.

#### List of candidate major gene(s) providing *S. phureja* cultivar i-0144787 with resistance to GPCN

**Fig. 3:**
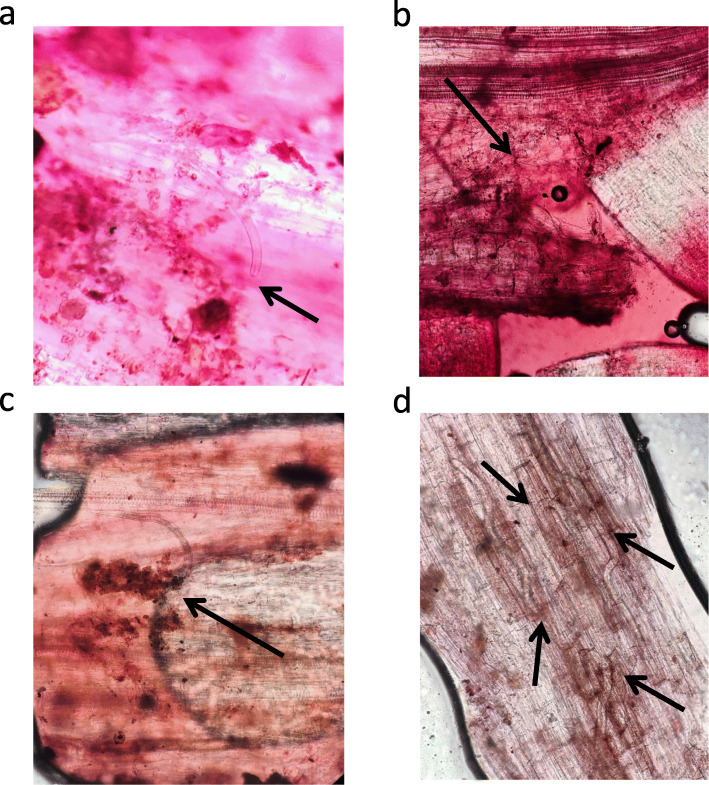
GPCN juvenile penetration into the root tissues of the susceptible *S. phureja accession* i-0144786 (**a**, 72 hpi) and resistant *S. phureja* accession i-0144787 (**b**, 24 hpi; **c**, 48 hpi; **d**, 72 hpi). **a**, no signs of necrosis near the nematode juvenile; **b**, zone of necrosis close to the GPCN juvenile head; **c**, large necrosis zone near the GPCN juvenile head; **d**, GPCN juveniles are surrounded by necrosis zones. Arrows mark the GPCN juveniles

One of the tasks of this research concerned the identification of R-genes in the genome of *S. phureja* i-0144787 accession providing high resistance against GPCN. According to the results obtained, the resistance is mediated through HR-response and programmed cell death (Fig. 3) commonly connected to NBS-LRR type receptors.

Thus, we compiled a list of potential R-genes based on the following assumptions: (i) candidate genes should belong to NBS-LRR group; (ii) the level of expression of candidate genes should be much higher in the roots of the resistant *S. phureja* genotype (preferably, with no expression in the susceptible genotype); (iii) the level of expression of candidate genes should be detected in the roots of the resistant *S. phureja* genotype before inoculation with water or GPCN juveniles.

The list of 419 potential R genes described earlier in (Jupe et al., 2013) [34] was taken for this analysis. The search for homology with *S. phureja* transcripts revealed 17 genes with generally up-regulated and 13 genes with down-regulated expression in the roots of the resistant genotype (Additional file 5). Some of these transcripts were used for qRT-PCR to verify the RNA-seq results (Table 4).

**Table 4:** The list of DEGs verified with qRT-PCR

Gene ID	Description (annotation)
PGSC0003DMG401007575	Cc-nbs-lrr resistance protein
PGSC0003DMG400029220	Tospovirus resistance protein A
PGSC0003DMG400006570	Tospovirus resistance protein C
PGSC0003DMG400009635	Gene of unknown function
DN73565c0g1t1	novel NBS-LRR encoding transcript
PGSC0003DMG400018428	Bacterial spot disease resistance protein 4

In most cases, the qRT-PCR results supported RNA-seq data and all these genes can be considered as primary candidates for further R-gene search (detailed information on qRT-PCR is available as Additional files 6 and 7).

## Discussion

In this research, two diploid potato specimens from different regions of South America were compared. It has been shown earlier that the accession i-0144786 was susceptible to GPCN, whereas the accession i-0144787 was highly resistant [4]. Interestingly, the resistance observed was not associated with the presence of known R genes widely distributed in *S. tuberosum* commercial varieties (*Gro1–4, H1*). Comparative analysis of i-0144786 and i-0144787 accessions revealed considerable differences in general composition of their transcriptomes as well as in the expressed sets of NBS-LRR-encoding genes [35]. Since these *S. phureja* genotypes from different regions of South America evolved differently, we conducted the detailed comparative study of their root transcriptomes to evaluate both the potential mechanisms of resistance to GPCN and genetic divergence at the root transcriptome level.

The results obtained supported the earlier observations on the considerable difference of these *S. phureja* genotypes (Figs. 2, 3). The molecular mechanisms associated with resistance to *G. rostochiensis* juveniles were further analyzed with the aid of AgriGO (Table 1). The experiments includes inoculation of potato plant roots with either water or GPCN juveniles and analysis of samples at 0 hpi (i.e., before inoculation), 24 hpi, and 72 hpi. Indeed, the process of root inoculation results in tissue mechanical wounding per se. Comparison of root transcriptomes of both resistant and susceptible genotypes before inoculation with water (0 hpi) and at 24 hpi revealed upregulation of cell cycle related terms whereas a wide variety of regulatory and metabolic processes were suppressed. This trend was even more pronounced at 72 hpi time point. It is likely that the transcriptome dynamics reflected potato root response on moderate wounding stress resulted from the experiment design and mostly associated with tissue regeneration after damage. Interestingly, the resistant genotype was characterized by considerably stronger response on water-inoculation induced wounding at 24 hpi point whereas such a difference with the susceptible genotype was not observed at 72 hpi (Table 1).

Inoculation of potato roots with GPCN results in a considerably stronger tissue damage because of juveniles penetration and movement in the root cylinder and vascular system. The changes in the transcriptome of the root cells of the susceptible genotype after GPCN inoculation was rather similar to water control: the AgriGO terms for up-regulated DEGs were mostly associated with cell cycle, mitosis (24 hpi, 72 hpi) and a variety of metabolic processes (72 hpi). Since the successful nematodes infestation results in the development of syncytia (feeding sites), the cell cycle and tissue regeneration related AgriGO terms may also reflect this process. The resistant *S. phureja* reacted to GPCN inoculation quite differently: at 24 hpi point, a variety of stress response related genes was up regulated. This list included terms, associated with oxidative stress, peroxidase activities, peptidase inhibitors, glycosylases. At 72 hpi, the root transcriptome of the resistant genotype reflected a strong response towards biotic stress and related processes (oxidation reduction, peroxidase activity, glycosylases, endopeptidases, proteases, ribonucleases, etc.). Activation of genes associated with tissue regeneration was also detected (cell wall biogenesis, cell division, etc., Table 1). It is likely that these terms may reflect regeneration of root tissues wounded by migrating GPCN juveniles as well as some other processes, e.g. initiation of syncytia, and regeneration after local programmed cell death because of a hypersensitive response. Thus, there is a strong difference in transcriptome dynamics in the roots of resistant and in the roots of susceptible *S. phureja* genotypes at 0, 24, and 72 hpi. Interestingly, the comparison of transcriptomes between resistant and susceptible genotypes appeared less informative, probably because of the genetic divergence between these accessions. Another observation pointed out the DEGs related to plastid-located metabolic chains. It is likely that processes in plastids may be of importance in establishing resistance and should be taken for further analysis.

In general, the resistant *S. phureja* genotype is characterized with a more pronounced response to wounding stress as well as a strong induction of biotic stress response genes at 24 hpi point. The resistance mechanism integrates the local hypersensitive response (Fig. 3) together with a systemic induction of defense-related genes (Table 1). GPCN infestation is accompanied with high expression of cell division related genes in both resistant and susceptible genotypes that may reflect tissue regeneration after juveniles’ movement and cell reprogramming by nematode secreted regulatory factors.

It was found that root transcriptomes of *S. phureja* accessions contained considerable parts of transcripts not annotated in the reference genome and potentially encoding proteins with functional Pfam-annotated domains (Table 2). Natural varieties of potato and other cultivated species are widely used as a source of new breeding traits. Here we demonstrate that de novo transcriptome assembly provides additional data for revealing R genes either improperly annotated or even absent in the reference genome (Table 3). Several NBS-LRR encoding transcripts characteristic for *S. phureja* resistant genotype were selected for further mapping of major GPCN resistance trait(s) (Table 4).

In this research two natural *S. phureja* specimens were selected because of their contrasting resistance to GPCN. It is likely that these specimens collected in distant regions of South America evolved under pressure of different pathogens varieties and they bear specific subsets of R genes. Transcriptome analysis provides valuable data on genetic mechanisms of pathogen resistance establishment and novel, potentially useful R genes for their introgression into the breeding program.

## Conclusion

The inoculation of diploid potato *S. phureja* with GPCN juveniles is accompanied with root wounding stress followed by activation of regeneration processes. In the roots of GPCN-resistant *S. phureja* genotype, the chain of molecular events initiates the hypersensitive response at the juveniles’ invasion sites and provides high-level resistance. It was also demonstrated that comparative transcriptomic analysis is a useful tool to reveal the resistance mechanisms and candidate R-genes. In particular, de novo transcriptome assembling highlights new functional gene variants and considerably extends the reference genome annotation.

## Methods

### Plant material

Two accessions of diploid cultivated species *S. phureja* k-11,291 (collected in Peru) and k-9836 (from Bolivia) were obtained from the VIR potato collection (each accession was represented by one clone (genotype) with the VIR INs i-0144787 and i-0144786, respectively). According to plastid SSRs data, these accessions have different haplotypes, indicating their different maternal origins [36, 37]. It was also found that genotype i-0144786 was susceptible to GPCN (Ro1), whereas i-0144787 was highly resistant to GPCN (Ro1) but contains no DNA markers of *Gro1–4* and *H1* (TG689, 239E4 left/Alu I, and Gro1–4) [4, 35].

### Nematode inoculation

A population of *G. rostochiensis* (pathotype Ro1) originated from Leningrad Region, Russia (Belogorka) was characterized previously [4]. The nematode population was propagated on the susceptible cultivar ‘Nevsky’. Cysts were extracted from soil by the flotation technique and stored at 4 °C. *S. phureja* plant preparation and their inoculation with GPCN were conducted as described earlier [35]. Infected roots, stained with acid fuchsin were scanned for the presence of nematodes under an AxioScope A1 light microscope (Carl Zeiss, Germany).

### RNA extraction

For RNA-seq, *S. phureja* roots were collected before inoculation (0 h), 24 and 72 h after inoculation with *G. rostochiensis* (pathotype Ro1). For each genotype, three infected and three control (water-inoculated) plants were taken. It should be noted that the nematode inoculation technique results in some root wounding, thus water-inoculated roots provide an important control for transcriptomes comparison. The roots of these plants were thoroughly rinsed with sterile distilled water, fixed in liquid nitrogen and used for RNA extraction. Total RNA was extracted with an RNeasy Plant Mini Kit (Qiagen).

### RNA-seq

The quality of the RNA samples was evaluated using a Bioanalyzer 2100 (Agilent), all samples had RIN 8.2 or higher. Small RNA and total RNA fraction was extracted using mirVana™ miRNA Isolation Kit (Ambion). RNA-seq library preparations were carried out with 1.5 mkg of total RNA fraction using TruSeq® Stranded mRNA LT Sample Prep Kit (Illumina) according to the manufacturer’s instructions for barcoded libraries with small modifications (4 min RNA fragmentation time and 12 PCR cycles were used). Final libraries quantification was performed with a Bioanalyzer 2100 and a DNA High Sensitivity Kit (Agilent). After normalization, barcoded libraries were pooled and sequenced on a NextSeq550 sequencer 2 × 150 bp using a High Density Cassette and NextSeq® 500 High Output v2 Kit 300 cycles (Illumina).

### qRT-PCR

RNA samples were treated with DNAse (QIAGEN RNase-Free DNase Set). To prepare single-stranded cDNA by reverse transcription, 1 μg aliquots of total RNA samples were used with a RevertAidTM kit (Thermo Fisher Scientific Inc., Waltham, MA, USA). Primers for qRT-PCR were designed with the aid if OLIGO software. β-tubulin gene was used as a reference (GenBank: Z33382; forward primer 5`-AGCTTCTGGTGGACGTTATG-3`, reverse primer 5`-ACCAAGTTATCAGGACGGAAGA-3`). The qRT-PCR was conducted using a SYNTOL SYBR Green I kit (Syntol, Moscow, Russia). For each reaction, three technical replicates were run.

### Bioinformatic analysis of RNA-seq data

#### Libraries pre-processing

Thirty libraries of paired-end short reads obtained with Illumina NextSeq550 sequencing platform were analyzed. Sequencing quality and length distributions were assessed using FastQC software (https://www.bioinformatics.babraham.ac.uk/projects/fastqc/) version 0.11.5. Cutadapt software version 1.9.1 [38] was used to remove sequencing adapters, and Prinseq-lite software version 0.20.4 [39] was implemented for filtering with the following criteria: length not less than 100 bases, mean Phred quality score not less than 30. In case if one read in a pair did not meet the above criteria, the whole pair was discarded from analysis. Additionally, Prinseq option ‘-derep 1’ was employed in order to remove possible optical replicates.

Libraries of short reads were aligned to the reference genome version SolTub_3.0 taken from Ensembl plants database [29] using STAR aligner version 2.5.3a [40].

#### Gene expression analysis

EdgeR package for R [41] was implemented to identify the differentially expressed genes (DEGs). First, tables of read counts were filtered to remove genes with expression levels below the threshold (not less than 3 counts per million for not less than 2 of 30 libraries). PlotMDS function of R was used to visualize multi-dimension clustering of the libraries based on the gene expression levels. Second, read counts were normalized using EdgeR function calcNormFactors. The dispersion was evaluated using estimateDisp function of EdgeR. The resulting matrix of pseudo-counts and dispersion values were subjected to glmQLFit function of EdgeR. Finally, glmQLFTest function was used to identify DEGs in the roots transcriptomes collected under different experimental conditions. In addition, read counts were normalized using TPM procedure, and mean TPM values were computed. Genes were classified as differentially expressing if a sum of mean TPM values for the experiments compared was not less than 10, changes in expression levels between experiments as computed by EdgeR were not less than two-fold (|log2(FC)| ≥ 1), and false discovery rate value was less than or equal to 0.05 (FDR ≤ 0.05).

Further, Gene Ontology terms enrichment analysis was performed using online tool ‘Singular Enrichment Analysis’ of AgriGO v2 [42] with default parameters (Fisher test with Yekutieli correction was applied, terms with *p* < 0.05 were considered significantly enriched). Analysis was conducted separately for groups of up-regulated and down-regulated DEGs.

#### De novo transcriptomes assembly

De novo assembly of *S. phureja* transcriptomes was performed using Trinity software version 2.6.5. Transcriptomes of two genotypes were assembled separately from 15 libraries corresponding to resistant or susceptible genotypes. Thus, two ‘master transcriptomes’ were assembled, each corresponding to one of the *S. phureja* genotypes. Further analysis of assembled transcriptomes included the following steps: redundancy reduction, transcript quantities analysis, genome representation search and Pfam-annotated domains prediction, including NBS and LRR domains prediction.

In order to reduce redundancy of assembled transcriptomes, tr2aacds.pl utility from Evidential Gene pipeline version ‘18may07’ was implemented. This utility identifies open reading frames (ORFs) in assembled contigs, and discards contigs lacking at least one ORF. Then it merges contigs together based on their predicted ORFs – if a sequence of one ORF of one contig is a subsequence of a longer ORF of another contig, the contig with shorter ORF is removed. Resulting sets of contigs were classified as non-redundant. Additionally, this utility identifies amino acid sequences of the proteins corresponding to the predicted ORFs.

BUSCO (Benchmarking Universal Single-Copy Orthologs) software version 3.0.2 [43, 44] was utilized to evaluate the quality of the assemblies. Non-redundant sets of contigs were subjected to BUSCO search against the embryophyta_odb9 database. BUSCO was designed specifically to assess genomes or transcriptomes quality.

To assess contig coverage by short reads of RNA-seq libraries, non-redundant sets of contigs were indexed using bowtie2-build utility (version 2.2.9) [45]. Then, several utilities of Trinity software package were applied to analyze the transcription levels of contigs. First, ‘abundance_estimate_to_matrix’ utility was used. Alignment-based abundance estimation approach was chosen and abundance values for each contig among all libraries were estimated with the aid of eXpress software version 1.5.1. The ‘filter_low_expr_transcripts’ utility was used to remove contigs with expression values below the chosen thresholds based on ‘Transcript Per Million’ (TPM) values. Three Trinity utilities were used to investigate the differential expression of the assembled contigs: ‘run_DE_analysis’, ‘DE_results_to_pairwise_summary’, ‘pairwise_DE_summary_to_DE_classification’. The ‘run_DE_analysis’ utility was run with previously obtained expression values matrix.

To compare the transcriptomes of *S. phureja* with the reference genome, Blat software version 34 [46] was used. In addition, with the aid of blastn utility of ncbi-blast standalone package version 2.7.1+ [47], non-redundant contigs were aligned to *S. tuberosum* CDS sequences version 3.0 obtained from Ensembl plants database. Corresponding predicted amino acid sequences of non-redundant contigs were aligned to *S. tuberosum* peptide sequences version 3.0 obtained from Ensembl plants database with the aid of blastp utility. For functional analysis and domain structure prediction, hmmscan utility of HMMER package (hmmer.org) version 3.1b2 was implemented. Pfam-A.hmm database of Pfam release 31.0 [48] was used as a reference database.

#### Prediction of de novo assembled transcripts potentially encoding NBS-LRR proteins

NLR-parser software, MEME suite version 4.9.1 [49] and MAST utilities were used for this purpose. Meme.xml file provided by Jupe and co-authors [34] was downloaded from GitHub repository and used as the motif definition file. The search for NBS-LRR motifs in predicted non-redundant amino acid sequences encoded by *S. phureja* transcriptomes was conducted using MAST. The resulting output file in .xml format was used as an input for NLR-parser software [33].

Particular cDNAs coding for NBS-LRR-like proteins with no homology to the reference genome were additionally analyzed. Nucleotide sequence of contigs were aligned to NCBI nr database using Nucleotide blast tool, and corresponding amino acid sequences were aligned to NCBI non-redundant protein sequences database. Ten best hits among protein sequences were used to create a multiple alignment file with ClustAl Omega web.

## Supplementary information

**Additional file 1.** File contains statistics of transcriptome libraries.

**Additional file 2.** File contains full lists of differentially expressed genes for all the experimental conditions.

**Additional file 3.** File contains full lists of GO terms for all the comparisons / experimental conditions.

**Additional file 4.** File contains the characteristics of de novo assembled transcripts.

**Additional file 5.** File contains the description of transcripts potentially encoding the NBS-LRR-related proteins.

**Additional file 6.** The description of gene-specific primers used for cDNA (qRT-PCR) amplifications.

**Additional file 7.** File contains the results of qRT-PCR validation of DEGs.

## Data Availability

Raw sequencing data is deposited in NCBI Sequence Read Archive as BioProject under accession number PRJNA515801 (https://www.ncbi.nlm.nih.gov/bioproject/PRJNA515801).

## Abbreviations

GPCN: Golden Potato Cyst Nematode
R gene: Resistance gene
VIR: Vavilov Institute of Plant Genetic Resources
DEGs: Differentially expressed genes
GO: Gene ontology
ORF: Open reading frame
BUSCO: Benchmarking Universal Single-Copy Orthologs
TPM: Transcripts per million
CDS: Coding DNA sequence
