# Supplementary material for: The mechanism of potato resistance to Globodera rostochiensis: comparison of root transcriptomes of resistant and susceptible Solanum phureja genotypes
Source: BMC Plant Biol. 2020 Oct 14;20(Suppl 1):350. doi: 10.1186/s12870-020-02334-2 (PMC7557027; doi:10.1186/s12870-020-02334-2)
Supplement: Supplementary file 6 — Additional file 6. The description of gene-specific primers used for cDNA (qRT-PCR) amplifications. [file 12870_2020_2334_MOESM6_ESM.docx]

Supplementary Table 1. Gene-specific primers used for gDNA (diagnostic) and cDNA (RT-qPCR) amplifications.

| **Gene** | **Purpose** | **PCR product length (bp)** | **Forward primer (5′→3′)** | **Reverse primer (5′→3′)** | **Annealing temperature. (ºC)** |
| --- | --- | --- | --- | --- | --- |
| PGSC003DMG401007575 | expression/ sequensing | 105 | GCTCTCACCAACTTCCAACTGCCT | ACCCCGAGCCACTGATGTTGAC | 60 |
|  | diagnostic | 78 | TTCAGCAAACTTGAAATATTGT | ACCCCGAGCCACTGATGT | 50 |
| PGSC0003DMG400029220 | expression/ sequensing | 129 | TGAAGCTGAGGTTGCCATTGA | ACCGGTCCCATCTCAATCAC | 60 |
| PGSC0003DMG400006570 | expression/ sequensing | 172 | ACTGGTTGAATGAGGCTATGG | TACGTCTCCACTTGTGTCTTC | 60 |
| PGSC0003DMG400009635 | expression/ sequensing | 201 | CTAACGGAGCAAAGGGAACT | GATTTAGTTCCTTTCACCAGCA | 60 |
| PGSC0003DMG400023288 | expression/ sequensing | 136 | GGTAGGTGGCAAGTGAATCC | CTGATGCCATAGGGAGTTTG | 60 |
|  | diagnostic | 127 | CAAGTGAATCCGCCAGCATC | - | 53 |
| PGSC0003DMG400018428 | expression/ sequensing | 88 | TGTGGAGAATAACTGTAGAGCAAAT | AGCTCCAACCCATCATAACTT | 60 |
| DN17537 | expression | 84 | GGAGCGTTACCAGTTTATTGC | TCGAGGAATGGAGGGTAGAT | 60 |
|  | sequencing/ diagnostic | 589 | - | AGACTTGATGGATTGGCAGAG | 55 |
