# Supplementary figures and images for: The mechanism of potato resistance to Globodera rostochiensis: comparison of root transcriptomes of resistant and susceptible Solanum phureja genotypes
Source: BMC Plant Biol. 2020 Oct 14;20(Suppl 1):350. doi: 10.1186/s12870-020-02334-2 (PMC7557027; doi:10.1186/s12870-020-02334-2)

PGSC003DMG401007575

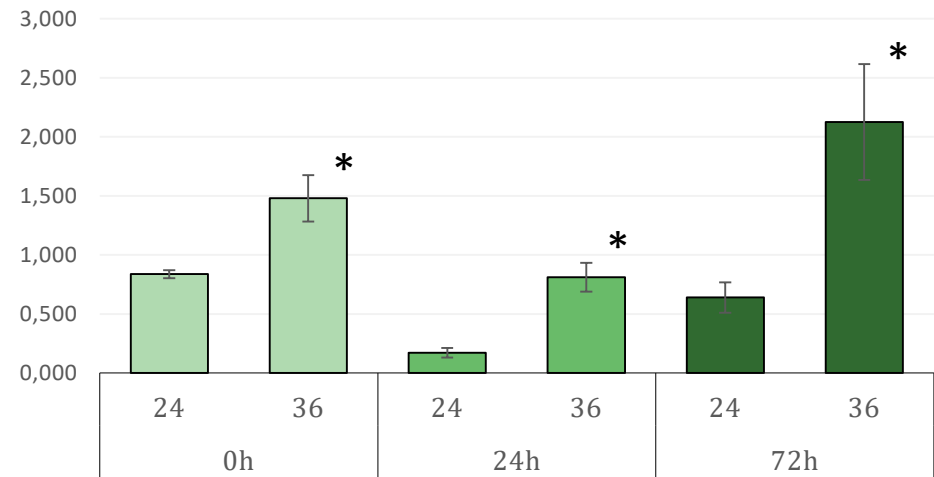

PGSC003DMG400029220

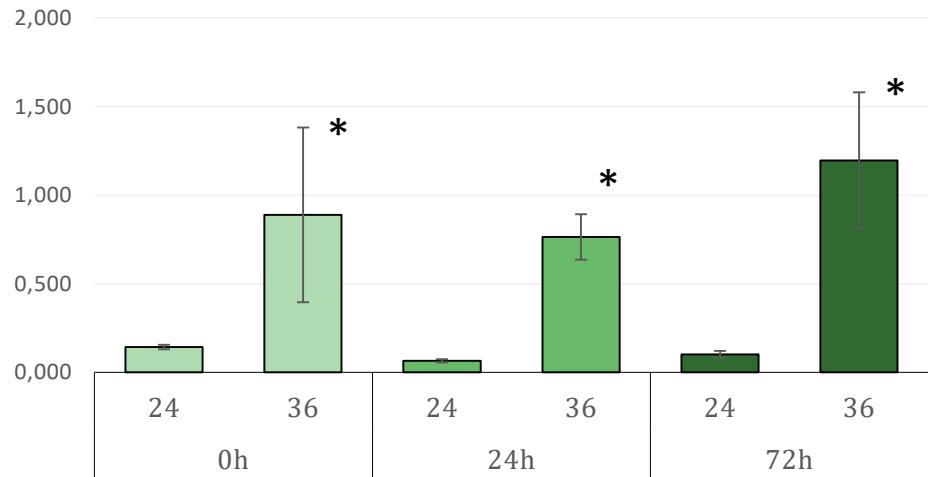

PGSC003DMG400006570

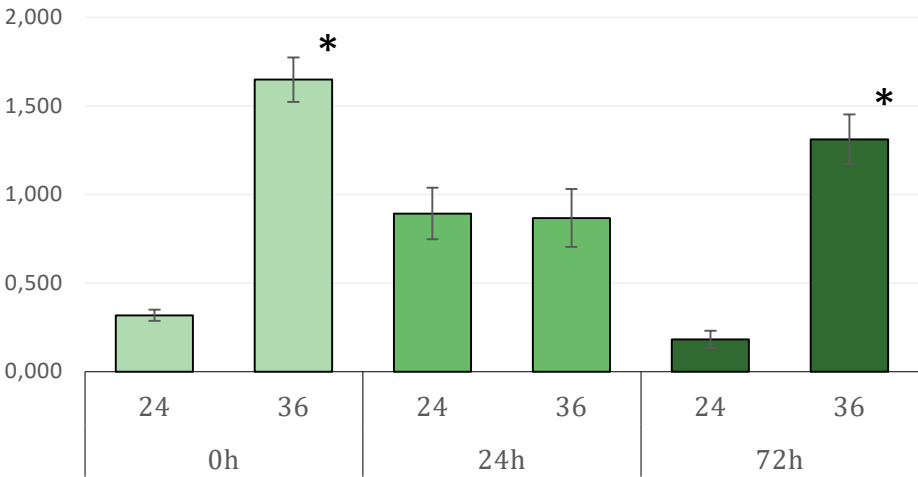

PGSC003DMG400009635

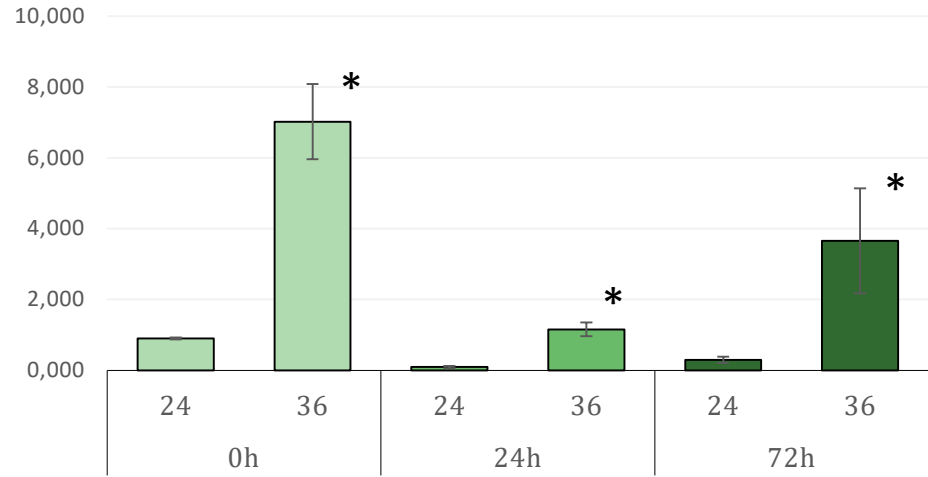

PGSC003DMG400023288

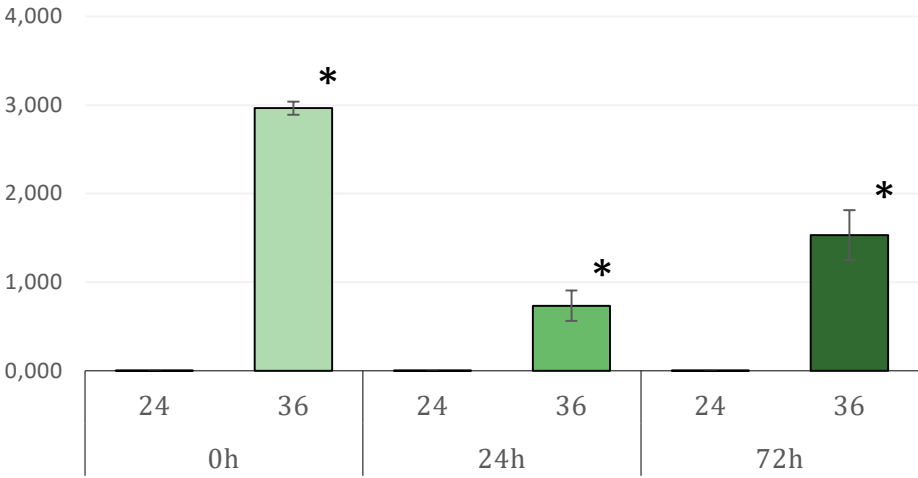

PGSC0003DMG400018428

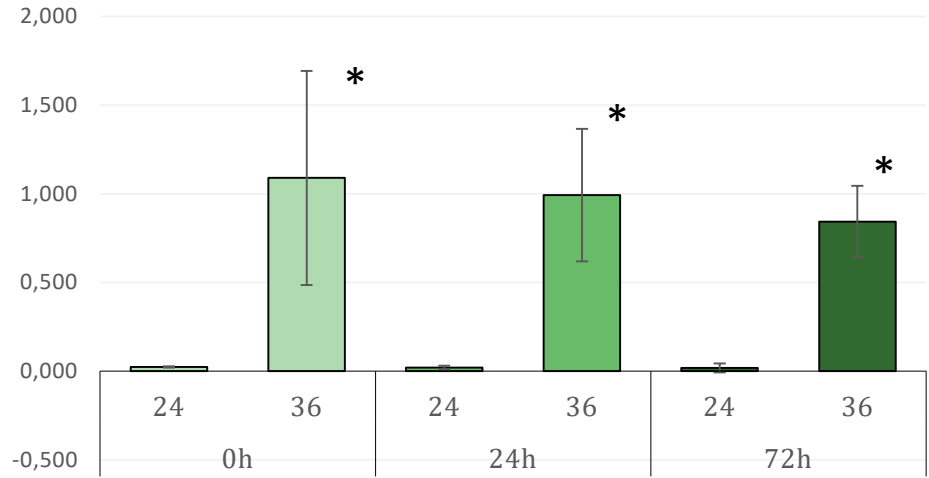

DN17537

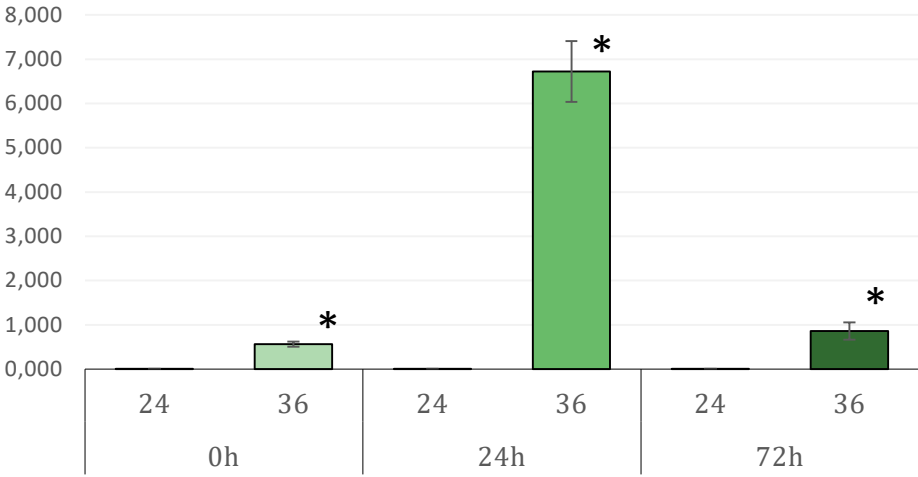

DN34164 DN39303 DN33041

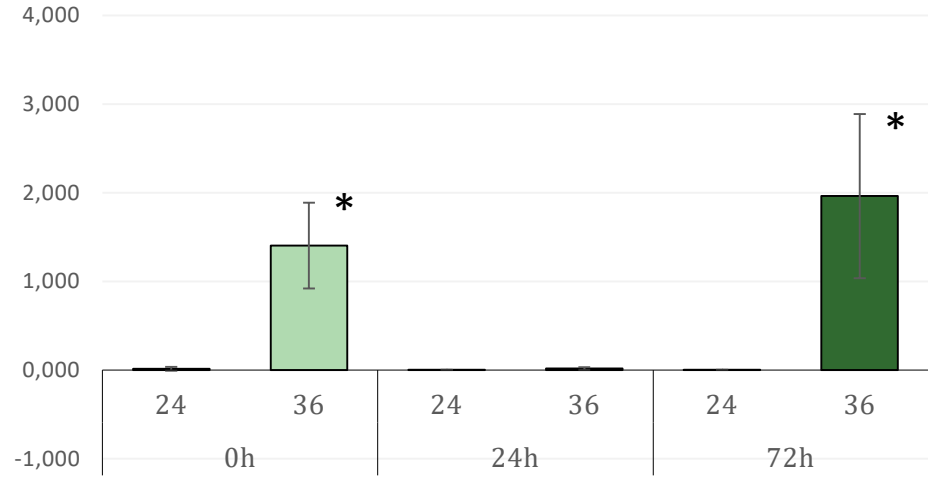

Supplement: Supplementary file 7 — Additional file 7. File contains the results of qRT-PCR validation of DEGs. [file 12870_2020_2334_MOESM7_ESM.pdf]
